# Aberrant right subclavian artery as soft marker in the diagnosis of trisomy 21 during the first trimester of pregnancy

**DOI:** 10.1007/s00404-021-06221-5

**Published:** 2021-09-22

**Authors:** Cristina Martínez-Payo, Elena Suanzes, Ana Gómez-Manrique, Alexandra Arranz, Tirso Pérez-Medina

**Affiliations:** 1 Prenatal Diagnosis Unit, Department of Obstetrics and Gynecology. Hospital, Universitario Puerta de Hierro Majadahonda, Calle Joaquín Rodrigo, 2. Majadahonda, 28222 Madrid, Spain; 2 Prenatal Diagnosis Unit, Department of Obstetrics and Gynecology, Hospital Universitario Puerta de Hierro Majadahonda, Madrid, Spain; 3 Prenatal Diagnosis Unit, Department of Obstetrics and Gynecology, Hospital Universitario Puerta de Hierro Majadahonda, Madrid, Spain; 4 Prenatal Diagnosis Unit, Department of Obstetrics and Gynecology. Hospital, Universitario Puerta de Hierro Majadahonda, Madrid, Spain; 5 Department of Obstetrics and Gynecology, Hospital Universitario Puerta de Hierro Majadahonda, Madrid, Spain

**Keywords:** Aberrant right subclavian artery (ARSA), First trimester, Trisomy 21, Aneuploidies, Prenatal ultrasound

## Abstract

**Purpose:**

Aberrant right subclavian artery is an anatomical variation with a prevalence of around 0.5–1.5% of the general population, being more frequently found among people with chromosomopathies, especially, trisomy 21. Despite being an anatomical finding, and thus, constant through the whole pregnancy, its value in the diagnosis of aneuploidies during the first trimester of pregnancy has been little studied. The aim of this study is to evaluate the reliability of the first-trimester ultrasound in the diagnosis of ARSA and its utility in the early diagnosis of aneuploidies.

**Methods:**

This was a descriptive, observational, cross-sectional study that included all fetuses with sonographic diagnosis of ARSA between 2011 and 2018.

**Results:**

There were 257 cases of ARSA diagnosed. The first-trimester ultrasound showed the following results in the detection of ARSA: sensitivity of 68% (CI 95% 60.8%–74.5%), specificity of 99.9% (CI 95% 99.9%–100%), positive predictive value of 93.7% (CI 95% 88.1%–96.8%), and negative predictive value of 99.6% (CI 95% 99.5%–99.7%). Due to the presence of ARSA, two cases of trisomy 21, that would have been missed in the first trimester, were diagnosed, using ARSA as a soft marker and modifying the risk obtained by the combined screening as part of the genetic sonogram of the first trimester.

**Conclusions:**

ARSA visualization during the first-trimester ultrasound is trustworthy and it can improve the detection of trisomy 21 in some cases of aneuploidy missed during the combined screening of the first trimester.

## Introduction

When from the aortic arch arise four branches instead of three, because the right subclavian artery directly arises from the aortic arch, it is called aberrant right subclavian artery (ARSA) [1]. ARSA usually passes behind the esophagus and the trachea, crossing from left to right, and though in the majority of the cases, it is asymptomatic, as a result of the compression of the mentioned organs; sometimes, it can cause dysphagia, cough, or dyspnea, what is called dysphagia lusoria [2]. ARSA is the most common abnormality of the aortic arch [3–6], and its prevalence in general population is between 1% and 1.5%. [7–9].

It is feasible to evaluate the aberrant right subclavian artery prenatally with ultrasounds, making use of the Color Doppler mode, since 12-week ultrasound and through the whole pregnancy [9]. It can be visualized if ARSA goes in front of or behind the trachea, approximately in 82%–84% of the cases in the first trimester, and in 95%–98% of the cases in the second trimester [8, 10, 11]. These images can be taken with the ultrasound performed transabdominally and/or transvaginally depending, in the first trimester, on the crown–rump length of the embryo and the body mass index of the mother [8]. Prenatally sonographic studies performed during the second trimester have demonstrated that ARSA is present in around 0.4%–1.5% of the chromosomally normal fetuses [7, 8, 12–15].

In 2005, Chaoui et al. [13] described that the presence of ARSA in fetuses affected of trisomy 21 (T21) was more frequently than in euploid fetuses, something that had already been described in the autopsies performed in people affected with this aneuploidy, in which ARSA was found with a prevalence between 19% and 36% [16–18]. It has been calculated that the average prenatal prevalence of ARSA in fetuses with T21 is of 23.6% in the second trimester (range 9.1%–37.5%) and between 7.8 and 33.3% in the first trimester [7]. There are multiple papers that have found that ARSA has a higher prevalence among fetuses with chromosomal abnormalities and/or congenital heart diseases and/or other sonographic abnormalities than in healthy fetuses [8, 9, 11, 13–15, 19–24].

The main objective of our study is to determine, in our experience, the utility to assess the presence or absence of ARSA in the first-trimester ultrasound, in the context of early diagnosis of chromosomal abnormalities, especially T21.

## Patients and methods

We designed a descriptive, observational, cross-sectional study which included all pregnant patients that attended our Prenatal Diagnosis Unit for sonographic evaluation, in any trimester of pregnancy between January of 2011 and December of 2018, and whose children were born in our hospital. Sonographic data were recorded in an independent and available database where the presence or absence of ARSA is gathered.

**Fig. 1:**
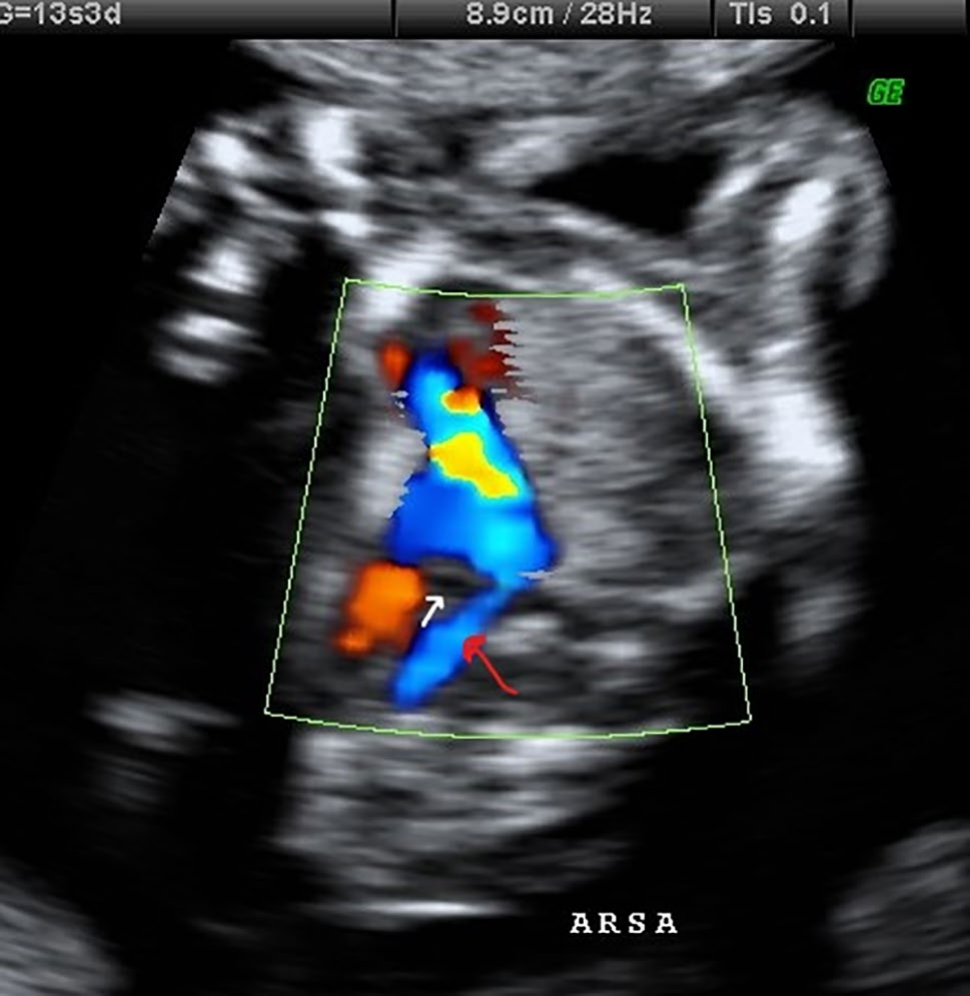
.

**Fig. 2:**
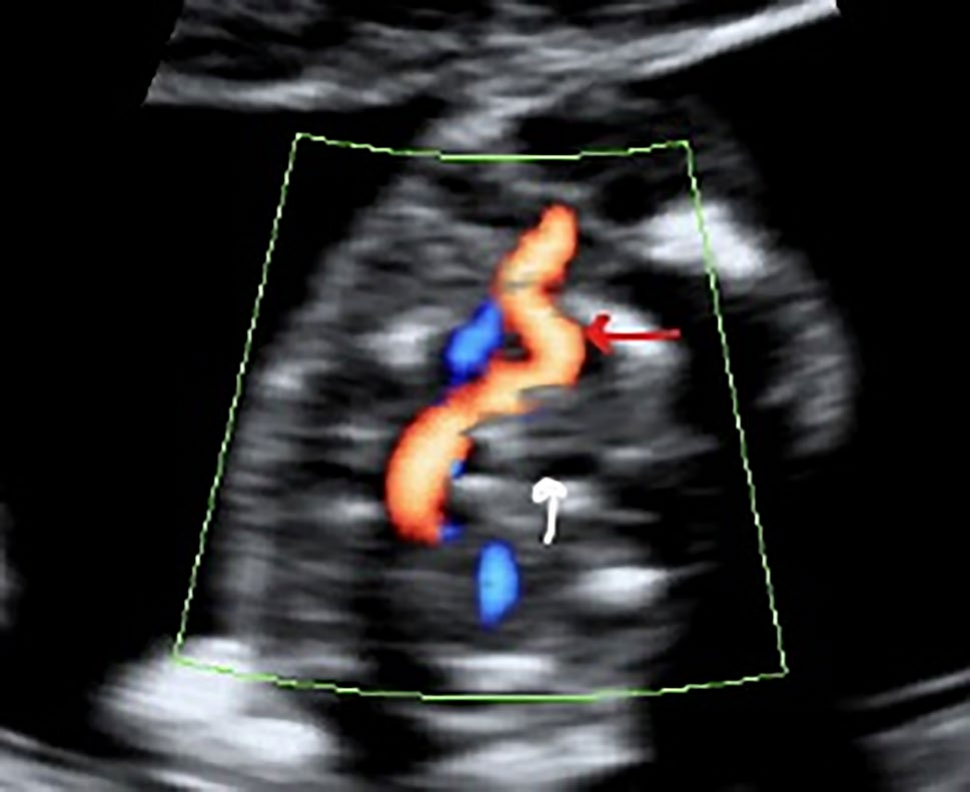
.

The ISUOG [25] and the Spanish Society of Obstetrics and Gynecology [26] recommend in the first trimester to evaluate the four heart chambers. Nevertheless, our team included as part of the routine clinical practice, in every trimester of pregnancy, the performance of the five views described by Yagel et al. [27] for the evaluation of the fetal heart, and since 2011, added the assessment of both subclavian arteries as a complementary sixth view. Right subclavian artery was identified in its origin in the aortic arch and in its relation with the trachea, using Color Doppler or Power Doppler when needed, and always following the criterion ALARA (As Low As Reasonably Achievable) recommended by the International Society of Ultrasound in Obstetrics and Gynecology (ISUOG) [25]. ARSA was considered when in a slightly more cephalad view than that one of the three vessels and trachea, a vessel was seen arising from the vertex of the V formed by the pulmonary artery and the aorta and directing toward right arm (Fig. 1). It is important to rule out the anatomical ante-tracheal location of right subclavian artery (Fig. 2), and to not confuse an ARSA with the azygos vein.

The ultrasounds were performed in most of the cases from a transvaginal approach in the first trimester and from a transabdominal one in the followings. All ultrasounds were conducted by specialists in prenatal diagnosis with more than 6 years of clinical experience. The equipment used was Voluson Expert 730 (GE Medical Systems, Zipf, Austria).

The screening of aneuploidies among general population in our center is performed through combined screening (CS), using the software PRISCA, 4.0.20.4 version (Siemens), which includes maternal age, analytical value of PAPP-A and β-hCG, and nuchal translucency in mm measured when the fetal crown–rump length is between 45 and 84 mm. We considered high risk of chromosomopathies when the software calculated a risk for T21 and/or T18 of 1/270 or more. Simultaneously, soft markers of aneuploidy such as ductus venosus flow velocities, tricuspid regurgitation, and the presence or absence of nasal bone were screened following the criteria of the Fetal Medicine Foundation [28]. The cases with intermediate risk of aneuploidy (between 1/271 and 1/1000) and any soft marker present turned out to be considered as high risk. In high-risk patients, an invasive complementary test was offered. The preferred one was chorionic villus sampling, until 2016 when non-invasive prenatal tests (NIPT) such as cell-free DNA were introduced to be implemented in a contingent way. In other words, after the performance of the combined screening (CS) with a result of high risk of aneuploidies or intermediate risk plus other positive soft marker, parents were given the chance to choose between a NIPT or an invasive one. In case of nuchal translucency ≥ 3.5 mm or P99, combined screening calculated risk ≥ 1/30, combined screening calculated risk between 1/31 and 1/270 with positive sonographic soft markers, major fetal malformation, or inheritable genetic disease, the patient was counseled to perform an invasive test better than a NIPT.

When we started assessing the presence of ARSA, the protocol described before did not change, but, when ARSA was suspected and considered an isolated finding, being the remaining parameters of the first-trimester ultrasound and the CS calculated risk average, another ultrasound in the week 16 was offered to confirm the diagnosis and to re-evaluate the case.

If the calculated risk was intermediate (between 1/271 and 1/1000), ARSA was confirmed in week 16 and it was an isolated finding, ARSA was considered then a positive soft marker, and parents were recommended to perform a genetic complementary test to rule out chromosomal abnormalities (amniocentesis or NIPT depending on the year and the will of the parents). Ultrasound scan was repeated in week 20 and fetal echocardiography was requested to the Pediatric Cardiology Unit with the intentions both to confirm the diagnosis and to rule out other accompanying heart defects. Sonographic follow-up was as well performed each 6 weeks until week 35 of pregnancy, re-evaluating the case and paying a special attention to the onset of new changes. Newborn was considered healthy when, after birth, pediatric evaluation considered so. Additionally, pediatric cardiologists performed at least one follow-up echocardiogram from the first month of age even in those cases considered isolated ARSA carriers.

True positives were considered when ARSA was identified in more than one ultrasound, excluding early ends of pregnancy due to TOP or miscarriage, where true positive was considered even with a single ultrasound.

For statistical analysis, we performed 2 × 2 tables including true-positive and true-negative results as well as false-positive and false-negative ones. These tables led us to calculate sensitivity, specificity, positive predictive value, and negative predictive value of the studied variables.

## Results

Between 2011 and 2018 in our unit, a total number of 279 fetuses were diagnosed of ARSA, out of a global of 16.589 fetuses that were tested for the presence or absence of this anomaly at any time during pregnancy. Among these 279 fetuses, 10 were considered false-positive results: eight in the first trimester and 2 in the second one. 12 cases were lost in follow-up. To sum up, 257 fetuses with ARSA were included in the study, which implied a prevalence of ARSA of 1.5% in our unselected population. From the total number of fetuses with diagnosis of ARSA, 175 were evaluated in the first trimester, and among them, in 119 the diagnosis of ARSA was checked in forward ultrasounds, and these cases were considered finally true-positive results (119/175). Thus, there were 56 cases in which ARSA was considered as not present in the first trimester, but its presence was identified in further ultrasounds, being these 56 cases considered false negatives (56/175) of the first trimester.

The evaluation of ARSA in the first trimester showed a sensitivity of 68% (CI 95% 60.8%–74.5%), a specificity of 99.9% (CI 95% 99.9%–100%), a positive predictive value of 93.7% (CI 95% 88.1%–96.8%), and a negative predictive value of 99.6% (CI 95% 99.5%–99.7%).

Nevertheless, studying the data year by year as we can see in Table 1, false-negative results tend to decrease as years go by, so that the detection rate of ARSA in 2018 rose to 90%.

**Table 1:** Year-by-year detection of ARSA in the first-trimester ultrasound

First trimester	2011	2012	2013	2014	2015	2016	2017	2018	Global
ARSA detected	5	15	8	13	18	27	12	21	119
ARSA not detected	7	13	7	6	8	7	6	2	56
%	41.7%	53.6%	53.4%	68.4%	69.2%	79.4%	66.7%	90%	68%

Table 2 shows the relation between ARSA and chromosomal abnormalities diagnosed in our unit through these years.

**Table 2:** Chromosomal abnormalities diagnosed during the study period

2011–2018	T21	T18	T13	Triploidy
Total diagnosticaded	91	39	8	3
Determined	63	22	4	–
ARSA Yes	10	2	1	1
ARSA No	53	20	3	–
%	16%	9%	25%	

Among the ten cases in which ARSA was present in fetuses affected by T21, ARSA’s finding was determining for the diagnosis of the aneuploidy in two of them: one case was an intermediate risk in the CS and isolated ARSA, and the other was a low risk in the CS but with other positive soft marker (hypoplastic nasal bone) apart from ARSA.

Twenty-five fetuses with ARSA showed intermediate risk in the CS (risk between 1/270 and 1/1000) and no other positive soft marker of the first trimester. There were no false-positive results in this group of patients. The probability to face a T21 in case of intermediate risk in the CS plus isolated ARSA is then of 4.8%.

There were four cases of ARSA and abnormal ductus venosus flow velocities in patients with low risk in the CS. Adding the case of T21 with low risk plus ARSA and hypoplastic nasal bone, the number of additional genetic tests performed due to the fact that the fetus showed an ARSA in the sonographic study was 30 tests in 8 years.

In case of T18, T13, and triploid fetuses, already since combined screening and week 12 ultrasound, important morphological abnormalities were found and/or high risk was calculated by CS, which made mandatory the performance of genetic prenatal tests, leaving ARSA in a second plane.

## Discussion

Nowadays, prenatal detection of the most frequent chromosomal abnormalities in the first trimester of pregnancy, when an adequate screening is performed, is high, so the number of fetuses that reach week 20 ultrasound misdiagnosed is actually very low. The use of soft markers in the first trimester added to the risk calculated with the combined screening improves even more the detection of such abnormalities, being the detection rate of 96% for a false-positive rate of 2.5% [29]. ARSA has been considered more as marker of the second trimester than of the first one. In the first trimester of pregnancy, ARSA has been little studied, even though the fact that being ARSA an anatomical malformation, it is always present throughout the whole pregnancy.

The attempt to view ARSA in the ultrasound of week 12 involves an effort that must be warranted. It took us, as a team, 7 years to reach the 90% of detection rate in the first trimester. In fact, ARSA’s finding was marginal in most of the cases of aneuploidy, because there were other data that guided us to the diagnosis of chromosomopathy. However, in two of the ten cases diagnosed of T21 where ARSA was found in the first trimester, this finding was determining for the diagnosis of the trisomy, or at least, enabled us to perform an earlier diagnosis: one case with intermediate risk in the CS and ARSA, and another with low risk, but with ARSA and hypoplastic nasal bone in the first trimester.

Hence, after the diagnosis of ARSA in week 12 of pregnancy, if it is a confirmed and isolated diagnosis in week 16, likelihood ratios described for the presence of ARSA in the second trimester [30] could be applied to the risk calculated by the combined screening in the first one. The mentioned risk applying these likelihood ratios would be four-folded. Another option would be considering ARSA as another soft marker of the first trimester such as ductus venosus flow velocities or tricuspid regurgitation are. In both ways of proceeding, the outcome is similar: any risk between 1/270 and 1/1000 will turn to be considered a high risk and we should recommend a complementary genetic test.

We think that the real utility of ARSA is in considering it an additional soft marker of the genetic sonogram of the first trimester, the same way the other soft markers are used in cases of intermediate risks in the CS; recommending complementary test even if it is an isolated finding, because the probability to be facing a T21 in this situation is 4.8% according to the data obtained from our patients. In case of having another positive soft marker additional to ARSA, it seems adequate to perform a complementary genetic test independently from the risk for T21 in the CS. This way of proceeding should not increase a lot the number of complementary tests to perform; according to our data, the increment would be less than 4 extra tests per year.

Another point to take in account is if detecting ARSA in the first trimester requires to wait for its confirmation in the echography performed 4 weeks later (in week 16). Due to the high specificity, and thanks to the possibility to perform NIPT, possibly this wait would not be necessary, something that will allow us to perform earlier diagnoses. Nevertheless, even if right subclavian artery did not seem aberrant in the first-trimester ultrasound, it should be checked again in week 20.

The relation between ARSA and other genetic abnormalities different from chromosomopathies is not clear. It seems that in case ARSA is not isolated finding throughout pregnancy sonographic follow-up, an invasive test to perform arrays might be indicated [31]. However, if ARSA remains as an isolated finding, the probability of pathologic findings in such arrays is minimal, and the invasive test may be avoided [11, 23, 31]. A more recent study on this topic found seven cases of abnormal microarrays among 133 cases of isolated ARSA, basically Di George Syndrome [32]. Considering these, parents should be properly informed and should as well take part in the decision whether to perform or not invasive tests.

The main limitations of the study are that there is no confirmation of ARSA in those fetuses that do not have the second-trimester ultrasound performed, because there are no necropsy studies, and that newborns considered healthy according to pediatric evaluation at birth have little follow-up from pediatric cardiologists. Probably, some cases of T21 in which ARSA was not detected were false negatives of the first trimester, and parents chose termination of pregnancy, missing the opportunity to diagnose an ARSA in forward ultrasounds at a more advanced gestational age. It is also possible that some cases that were not tested for ARSA, carried it. Nevertheless, the fact that we have found ARSA in 1.5% of our unselected fetuses, which, indeed, matches with the published data of sonographic evaluation of this anatomical variation [11], drives us to think that we are close to reality. Moreover, we have found a prevalence of ARSA in fetuses affected of T21 of 16% which also meets with the range published in the meta-analysis of Scala et al. [7].

We conclude saying that in our experience, ARSA shows a limited value in the diagnosis of aneuploidies in the first trimester of pregnancy, although it could help to increase the detection of such chromosomopathies before week 20 if it is implemented as a soft marker, modifying the risk calculated by the combined screening.
